# Alpha lipoic acid attenuates ER stress and improves glucose uptake through DNAJB3 cochaperone

**DOI:** 10.1038/s41598-020-77621-x

**Published:** 2020-11-24

**Authors:** Abdoulaye Diane, Naela Mahmoud, Ilham Bensmail, Namat Khattab, Hanan A. Abunada, Mohammed Dehbi

**Affiliations:** 1 Diabetes Research Center, Qatar Biomedical Research Institute (QBRI), Hamad Bin Khalifa University (HBKU), Qatar Foundation (QF), Doha, Qatar; 2 College of Health and Life Sciences, Hamad Bin Khalifa University, Qatar Foundation, Doha, Qatar

**Keywords:** RNA editing, RNA

## Abstract

Persistent ER stress, mitochondrial dysfunction and failure of the heat shock response (HSR) are fundamental hallmarks of insulin resistance (IR); one of the early core metabolic aberrations that leads to type 2 diabetes (T2D). The antioxidant α-lipoic acid (ALA) has been shown to attenuate metabolic stress and improve insulin sensitivity in part through activation of the heat shock response (HSR). However, these studies have been focused on a subset of heat shock proteins (HSPs). In the current investigation, we assessed whether ALA has an effect on modulating the expression of DNAJB3/HSP40 cochaperone; a potential therapeutic target with a novel role in mitigating metabolic stress and promoting insulin signaling. Treatment of C2C12 cells with 0.3 mM of ALA triggers a significant increase in the expression of DNAJB3 mRNA and protein. A similar increase in DNAJB3 mRNA was also observed in HepG2 cells. We next investigated the significance of such activation on endoplasmic reticulum (ER) stress and glucose uptake. ALA pre-treatment significantly reduced the expression of ER stress markers namely, GRP78, XBP1, sXBP1 and ATF4 in response to tunicamycin. In functional assays, ALA treatment abrogated significantly the tunicamycin-mediated transcriptional activation of ATF6 while it enhanced the insulin-stimulated glucose uptake and Glut4 translocation. Silencing the expression of DNAJB3 but not HSP72 abolished the protective effect of ALA on tunicamycin-induced ER stress, suggesting thus that DNAJB3 is a key mediator of ALA-alleviated tunicamycin-induced ER stress. Furthermore, the effect of ALA on insulin-stimulated glucose uptake is significantly reduced in C2C12 and HepG2 cells transfected with DNAJB3 siRNA. In summary, our results are supportive of an essential role of DNAJB3 as a molecular target through which ALA alleviates ER stress and improves glucose uptake.

## Introduction

Increased insulin resistance (IR) in peripheral organs and progressive decline in β-cell function are the two early and crucial pathophysiological aberrations leading to chronic hyperglycemia and overt type 2 diabetes (T2D). The etiology of the disease is complex and involves a complex interplay between genetic susceptibility and the negative health effects of a wide range of environmental and lifestyle factors, including high dietary fat content, physical inactivity, sedentariness and obesity^1^. Of these, obesity represents a significant contributing factor to T2D through the development of IR^2^.

During the course of obesity, several stress/inflammatory pathways, with adverse effects on the insulin receptor signaling are activated in metabolically relevant tissues, leading to disruption of systemic metabolic homeostasis^3^. Persistent ER stress^4^, inflammatory response^5^ and enhanced oxidative stress^6^, together with defects in mitochondrial function^7^, heat shock response (HSR)^8^ and the antioxidant defense system^9^ are the key hallmarks of IR and T2D. This metabolically toxic environment resulting from a tilted balance toward the pro-stress state leads to the activation of several kinases; particularly JNK1 stress kinase and IKKβ inflammatory kinase, which phosphorylate IRS1 on specific inhibitory serine residues and results in impaired insulin-mediated downstream signaling^10,11^. Therefore, therapeutic approaches that mitigate metabolic stress and promote the antioxidant and HSR have been proven to be effective in improving insulin sensitivity and glycemic control.

α-Lipoic acid (ALA), also called thioctic acid or 1,2-dithiolane-3-pentanoic acid is a naturally occurring dithiol compound enzymatically synthesized from octanoic acid in the mitochondria with a powerful antioxidant property. It acts as a crucial cofactor of the mitochondrial α-ketoacid dehydrogenase complexes involved in carbohydrates metabolism^12^. Beside its primary role as a cofactor, ALA elicits other biochemical activities such as scavenging free radicals, regenerating the cellular antioxidant agents such as GSH, vitamin C and E, and modulating several critical signal transduction pathways^13,14^. ALA is a commonly used and readily available dietary supplement. In humans and experimental animal models, administration of ALA proved its pharmacotherapeutic value with a great therapeutic window index against several chronic diseases associated with metabolic stress such as diabetes^15^, neuropathy^16^, obesity^17^, non-alcoholic fatty liver disease^18^, neurodegeneration^19^ and other vascular diseases^20^. The therapeutic use of ALA in improving insulin sensitivity and promoting glucose metabolism has been well documented both in humans and animal models of obesity associated with IR and T2D^15,21^. Interestingly, administration of ALA inhibited JNK and IKKβ activation, reduced the phosphorylation of IRS1 at the inactivating serine 307 residue, enhanced the PI3K/AKT pathway and consequently, stimulated both basal and insulin-mediated translocation of glucose transporters to the plasma membrane^22–24^.

The underlying molecular mechanisms by which ALA mediates those beneficial effects are not yet fully elucidated, but the implication of several components of the HSR has been documented^25–27^. Recent data from our laboratory demonstrated the impaired expression of DNAJB3; a member of DNAJ/HSP40 cochaperone family in obese^28^ and in T2D patients^29^, and that low levels of DNAJB3 were associated with enhanced metabolic stress^28^. We further demonstrated the positive effect of a 3-month physical exercise in restoring the normal expression of DNAJB3 with the concomitant improvement of various physical, biochemical and clinical parameters, suggesting thus a protective role of DNAJB3 against metabolic diseases associated with increased IR^28^. More recently, we provided evidence for a novel role of DNAJB3 in attenuating various forms of metabolic stress as well as in promoting insulin action and glucose uptake in 3T3-L1 adipocytes and C2C12 skeletal muscle cells^29,30^. Given the similarities in metabolic actions elicited by DNAJB3 overexpression and upon ALA treatment, we hypothesized that DNAJB3 might represent a molecular intermediate through which ALA mediates its beneficial actions.

The purpose of this investigation is therefore to assess the effect ALA treatment on the endogenous expression of DNAJB3 in metabolically relevant cells and determine the significance of such an effect on other forms of metabolic stress that trigger IR as well as on glucose uptake. We show that ALA triggers a significant increase in the expression of DNAJB3 in C2C12 and HepG2 cells. Given the modulatory effect of ALA on ER stress and glucose uptake, we next investigated the significance of such activation on ER stress; one of the key hallmarks of obesity induced IR and T2D. ALA pre-treatment significantly reduced the expression of ER stress markers namely, GRP78, XBP1, sXBP1 and ATF4 in response to tunicamycin. In functional luciferase-based assays, ALA treatment abrogated significantly the tunicamycin-mediated transcriptional activation of ATF6 while stimulating insulin-stimulated glucose uptake. Interestingly, knocking down the expression of DNAJB3 with siRNA abolished the protective effect of ALA on tunicamycin-induced ER stress, suggesting thus that DNAJB3 is a key mediator of ALA-alleviated tunicamycin-induced ER stress. Furthermore, the effect of ALA on insulin-stimulated glucose uptake was reduced significantly in C2C12 and HepG2 cells transfected with DNAJB3 siRNA. Together, our results suggest that DNAJB3 is a molecular target through which ALA alleviates ER stress and improves glucose uptake.

## Materials and methods

### Reagents

Anti-DNAJB3 antibody was purchased from Proteintech (Proteintech Group, Inc., Chicago, IL). Anti-HSP72 antibody was purchased from ENZO (ENZO Life Sciences, Inc., Farmingdale, NY). Anti-GRP78 and anti-γ-Tubulin were purchased from Abcam (Abcam, Cambridge, UK). Anti-GAPDH, Actin and horseradish peroxidase-conjugated antibodies were purchased from Cell Signaling (Cell Signaling Technology, Inc., Danvers, MA). Anti-HA Tag Alexa Fluor^®^ 647-conjugated Monoclonal Antibody was purchased from R&D Systems (R&D Systems, Abingdon UK). ALA, tunicamycin, H_2_O_2_ and insulin were purchased from Sigma (Sigma-Aldrich, St. Louis, MO). C2C12 and HepG2 were purchased from ATCC (ATCC, Manassas, VA). Fluorescently labeled d-glucose analog (2-NBDG) was purchased from Cayman (Cayman, Ann Arbor, MI). pHA-Glut4-GFP was a gift from Dr. MacGraw (Weill Cornell University, New York, NY) and consists of an exofacial HA epitope and a GFP tag located at the N-terminal and C-terminal of Glut4, respectively^30^. Reporter plasmid carrying five copies of ATF6 binding site upstream of the Luc2P reporter gene was described previously^30^. Scrambled and specific siRNA were purchased from Dharmacon (Dharmacon Inc., Lafayette, CO). Lipofectamine 3000 and lipofectamine RNAiMAX were purchased from Invitrogen (Invitrogen, Carlsbad, CA). Bright Glo Luciferase Assay kit was purchased from Promega (Promega Corporation, Madison, WI). PureLink™ RNA Minikit and High-Capacity cDNA Reverse Transcription Kit were purchased from Invitrogen (Invitrogen, Carlsband, CA). MitoTracker Red CM-H2XRos was purchased from ThermoFisher (ThermoFisher, Waltham, MA).

### Cell culture silencing RNA and transient transfections

C2C12 and HepG2 cells were maintained in DMEM supplemented with 10% FBS and 1% penicillin/streptomycin at 37 °C and 5% CO2. Differentiation of C2C12 from myoblasts to myotubes was done by replacing FBS with 2% horse serum with a daily change of the media for 5 days. For heat shock induction, ~ 85% confluent cells were incubated for 1 h at 43 °C followed by a 4 h recovery at 37 °C and then harvested. For the effect of ALA on ER stress, cells were treated with either ALA (0.3 mM) or vehicle for 24 h and thereafter, ER stress was induced with tunicamycin at a dose of 0,5 μg/ml for overnight afterwards, harvested for RNA and proteins extraction.

Lipofectamine 3000 and RNAiMAX lipofectamine were used for transient DNA and siRNA transfection, respectively as recommended by the manufacturer. We used a smart pool Accell siRNA targeting mouse DNAJB3 (E-046394-00-0005), human DNAJB3 (E-032304-00-0005), mouse HSP72 (E-054644-00-0005), human HSP72 (E-005168-01-0005) and Accell non-targeting control “scrambled” (D-001910-10-05). All siRNAs were used at 20 nM as recommended by the manufacturer. The sequences of the siRNAs used in this study are listed in Table 1. All the functional assays were analyzed at least in triplicate and a minimum of three independent experiments.

**Table 1:** List of siRNAs and sequences.

Species/genes	Dharmacon references	Target sequence
Mouse/Dnajb3	1. A-046394-13	CCGAAAUAAUUAAUGGCAA
	2. A-046394-14	UUAAAGUCCCUGAUAAUUA
	3. A-046394-15	CAGGCAACUACAAGUCGGU
	4. A-046394-16	GGCUUCGCUUCGUUAGAUA
Human/Dnajb3	1. A-032304-13	CCUUUGACCUCUUGGGAAA
	2. A-032304-14	CAUUUGACUUUAUUGUUUA
	3. A-032304-15	GCUGUACCCAAGAAUUUAU
	4. A-032304-16	CUGAAAUAGUUGAUGGUAA
Mouse/HSP72	1. A-054644-13	CCGCUGAUGUGAUUUGUUU
	2. A-054644-14	UUAUCUUCCCUGUUAAUUA
	3. A-054644-15	CUGUCAUUAUUUCAAGUUU
	4. A-054644-16	CUUUCAGUUACUUUGUGUA
Human/HSP72	1. A-005168-14	CUAGUAUUUCUGUUUGUCA
	2. A-005168-15	CUGCCAUCUUACGACUAUU
	3. A-005168-17	CCUGUGUUUGCAAUGUUGA
	4. A-005168-18	CCAUUGAGGAGGUAGAUUA
Scrambled control	D-001910-10-05	UGGUUUACAUGUCGACUAA
	D-001910-10-05	UGGUUUACAUGUUUUCUGA
	D-001910-10-05	UGGUUUACAUGUUUUCCUA
	D-001910-10-05	UGGUUUACAUGUUGUGUGA

### Luciferase assays

C2C12 and HepG2 were grown in 100 mm petri dish and at ~ 80% confluence, they were transfected with 7.5 µg of the reporter plasmid using lipofectamine 3000 and incubated overnight at 37 °C. On next day, they were plated on 96-well plates at 1 × 10^4^ cells/well in complete DMEM media containing 0.3 mM ALA or vehicle. After 8 h of incubation, cells were stimulated with 0.5 µg/ml tunicamycin or vehicle and incubated at 37 °C for overnight and then, harvested for luciferase assays using the Bright Glo Luciferase Assay kit as we described previously^30^. To investigate if the effect of ALA on ER stress is mediated through DNAJB3, knocking down DNAJB3 in C2C12 cells was carried out with DNAJB3 siRNA or scrambled siRNA using lipofectamine RNAiMAX protocol. The following day, cells were transfected with 7.5 μg ATF6 reporter plasmid using Lipofectamine 3000 and incubated overnight at 37 °C. Afterward, cells were then plated in 96-well plate at 1 × 10^4^ cells/well and pre-treated with ALA for 24 h followed by overnight tunicamycin stimulation and luciferase activity as described above.

### Measurement of gene expression by real-time RT-PCR

RNA was extracted from treated cells using PureLink™ RNA Minikit as instructed by the manufacturer. It was then converted to cDNA using High-Capacity cDNA Reverse Transcription Kit and analyzed by RT-PCR on QuantStudio 6 Flex system using SYBR Green (ThermoFisher, Waltham, MA). Relative expression was calculated by the comparative ΔΔCt method^31^. Briefly, relative gene expression was calculated by the difference (ΔCt) between the Ct value of the gene of interest and that of the reference gene. Then the difference in the delta value between the experimental and control group (ΔΔCt) and the fold change (2^−ΔΔCt^) were calculated. GAPDH and actin genes were used as internal controls. The primers corresponding to genes of the heat shock response, ER stress and oxidative stress have been published previously^30,32^. The ones used for mitochondria were self-designed using Primer 3 software version 4.0 (http://bioinfo.ut.ee/primer3-0.4.0). The sequences of the primers are listed in Table 2.

**Table 2:** Primer list and sequences.

Gene	Forward	Reverse
DNAJB3	5′-AGGGGCTGTACCCTTCTCTA-3′	5′-AGTTTCCTGGAGAACCGAAG-3′
SOD1	5′-GAGAGGCATGTTGGAGACCT-3′	5′-CCACCTTTGCCCAAGTCATC-3′
Catalase	5′-AGGAGGCAGAAACTTTCCCA-3′	5′-GGCCCTGAAGCATTTTGTCA-3′
GPX1	5′-ATCAGTTCGGACACCAGGAG-3′	5′-GATGTACTTGGGGTCGGTCA-3′
ATF4	5′-GGGTTCTGTCTTCCACTCCA-3′	5′-AAGCAGCAGAGTCAGGCTTTC-3′
GRP78	5′-AATTTCTGCCATGGTTCTCA-3′	5′-AGCATCTTTGGTTGCTTGTC-3′
XBP1	5′-TCCCCAGAACATCTTCCCAT-3′	5′-ACATGACAGGGTCCAACTTG-3′
sXPB1	5′-CTGAGTCCGAATCAGGTGCAG-3′	5′-GTCCATGGGAAGATGTTCTGG-3′
HSF1	5′-GCTCAACATGTATGGCTTCC-3′	5′-GCTGGTCACTTTCCTCTTGA-3′
HSP70	5′-TCTCCTGTCTTGTCCGAGAG-3′	5′-ATGCTGACTTGACCTTGAGC-3′
HSP72	5′-GACAAGAAGAAGGTGCTGGA-3′	5′-TGGTACAGCCCACTGATGAT-3′
HSP90	5′-TGAAACTGCTCTGCTCTCCT-3′	5′-CTCCTCTGCAGTGACCTCAT-3′
PGC1α	5′-CACCAAACCCACAGAAAACAG-3′	5′GGG TCAGAGGAAGAGATAAAGTTG-3′
TFAM	5′-GCTTGGAAAACCAAAAAGAC-3′	5′-CCCAAGACTTCATTTCATT-3′
PPARγ	5′-GATGTCTCACAATGCCATCAG-3′	5′-TCAGCAGACTCTGGGTTCAG-3′
PPARα	5′-AACATCGAGTGTCGAATATGTGG-3′	5′-CCGAATAGTTCGCCGAAAGAA-3′
Cytochrome C	5′-CTGTGGAAAAGGGAGGCAAG-3′	5′-CACCTGGTAATTCTGCACTGG-3′
GAPDH	5′-CTGGAGAAACCTGCCAAGTA-3′	5′-AGTGGGAGTTGCTGTTGAAG-3′
Actin	5′-AAGAGCTATGAGCTGCCTGA-3′	5′-GATGCCACAGGATTCCATAC-3′

### Measurement of mitochondrial activity

In order to determine mitochondrial content, 1 × 10^4^ of C2C12 cells were seeded in quadruplicate in a 12-well plate containing coverslips. They were then treated with 0.3 mM of ALA or vehicle for 24 h and thereafter; mitochondria were labelled with 500 nM of MitoTracker Red CM-H2XRos for 30 min before visualization. DAPI was used as a counter screen to visualize nuclei. Images were acquired using a 25 ×/0.8 numerical aperture (NA) objective (LD LCI Plan‐Apochromat; Carl Zeiss Inc., Oberkochen, Germany) using confocal microscopy on a laser scanning microscope (LSM 780; Carl Zeiss Inc., Oberkochen, Germany), mounted on a laser scanning microscope (LSM) (Zeiss LSM 780; Carl Zeiss Inc.,). Images were analyzed using ZEN imaging software (Carl Zeiss Inc.). The fluorescence intensity (40 cells in the vehicle group and 41 cells in the ALA group) was quantified using ImageJ 1.52v software (NIH, Bethesda, MA, USA) and the bar graphs data are mean ± sem and presented as fold change.

### Preparation of whole cell lysates and western blot analysis

Whole protein extracts were prepared from treated cells using RIPA buffer (50 mM Tris·HCl, pH 7.5, 150 mM NaCl, 1% Triton X-100, 1 mM EDTA, 0.5% sodium deoxycholate and 0.1% SDS). Protein concentration was determined by the Bradford method using γ-globulin as a standard, and 40 µg of proteins were resolved on 10% SDS-PAGE gels and used to detect GRP78, GAPDH and γ-Tubulin, while for DNAJB3 and HSP72, 80 µg of proteins were loaded. Proteins were then transferred onto PVDF membranes, blocked with 5% nonfat dried milk in Tris-buffered saline containing 0.05% Tween 20 (TBST) for 1 h, and then probed with the primary antibody for overnight at 4 °C. GAPDH, Actin and γ-Tubulin were used as internal controls. Antibodies recognizing DNAJB3, HSP72 and GRP78 were used at dilutions of 1:500, 1:500 and 1:1000, respectively. Anti-GAPDH, anti-actin and anti-γ-Tubulin antibodies were used at dilutions ranging from 1:1000 to 1:3000. After washing, the membranes were incubated with horseradish peroxidase-conjugated secondary antibody at a dilution of 1:2000 for 2 h at room temperature. Protein bands were visualized by chemiluminescence and the images were captured using the ChemiDoc XRS^+^ system (Bio-Rad, Hercules, CA). For densitometric analysis, the intensity of the bands was determined using ImageJ 1.52v software (NIH, Bethesda, MA, USA).

### Glucose uptake assay

C2C12 and HepG2 cells at 80% confluence in 100 mm petri dishes were pre-treated with either 0.3 mM ALA or vehicle for 8 h and then, the media was replaced with glucose free media, containing either ALA or vehicle, for overnight starvation. The next day, the culture media was replaced with glucose free culture media containing Fluorescent tagged d-glucose analog (2-NBDG) at a concentration of 150 μg/ml with or without 100 nM of insulin and incubated for 1 h at 37 °C. Cells were then washed with Cell-Based Assay Buffer and transferred to 96-well assay plate at 1 × 10^4^ cells/well. Finally, fluorescence was quantified on Glomax Multi^+^ Microplate Multimode Reader (Promega, Madison, WI) as previously described^29,30^. Monitoring glucose uptake in C2C12 and HepG2 cells where the expression of DNAJB3 has been silenced was done by transfecting cells with DNAJB3-siRNA or scrambled control and after 2 days, cells were pre-treated with either 0.3 mM ALA or vehicle for 8 h and the media was changed to glucose free media with either ALA or vehicle for starvation and glucose uptake was carried out as described above.

### Monitoring of Glut4 translocation by flow cytometry

C2C12 cells at 80% confluence in 6-well plates at 200,000 cells/well were transfected with 3.75 µg of HA-Glut4-GFP plasmid. The following day, cells were pre-treated with either 0.3 mM ALA or vehicle for 24 h. After glucose and serum starvation for 1 h, cells were stimulated with 100 nM of insulin for 1 h, trypsinized, collected in falcon tubes (100,000 cells/tube) and stained with a mouse Anti-HA Tag Alexa Fluor^®^ 647-conjugated Monoclonal Antibody for 30 min. Thereafter, cells were washed 2 times with FBS stain buffer and the HA Tag Alexa Fluor 647 (labelling only the Glut4 on the plasma membrane) and GFP (reflecting the total Glu4 expression) positive cells were determined by flow cytometry. The surface-to-total Glut4 ratio (HA/GFP) was calculated to determine the Glut4 translocation.

### Statistical analysis

All assays were performed at least in triplicate and a minimum of three independent experiments.

Results are presented as means ± SEM and were plotted using GraphPad (Prism v7, La Jolla, CA). Shapiro–Wilk test was first performed for the normality test followed by parametric tests. We used unpaired *t* tests or two-way analysis of variance to test gene (Scrambled- vs SiRNA) by treatment condition (vehicle vs treatment) effects and one-way analysis of variance for comparison of the groups with post-hoc Tukey’s test for pairwise comparisons. A P-value < 0.05 was considered statistically significant.

## Results

### ALA induces the endogenous expression of DNAJB3

**Figure 1:**
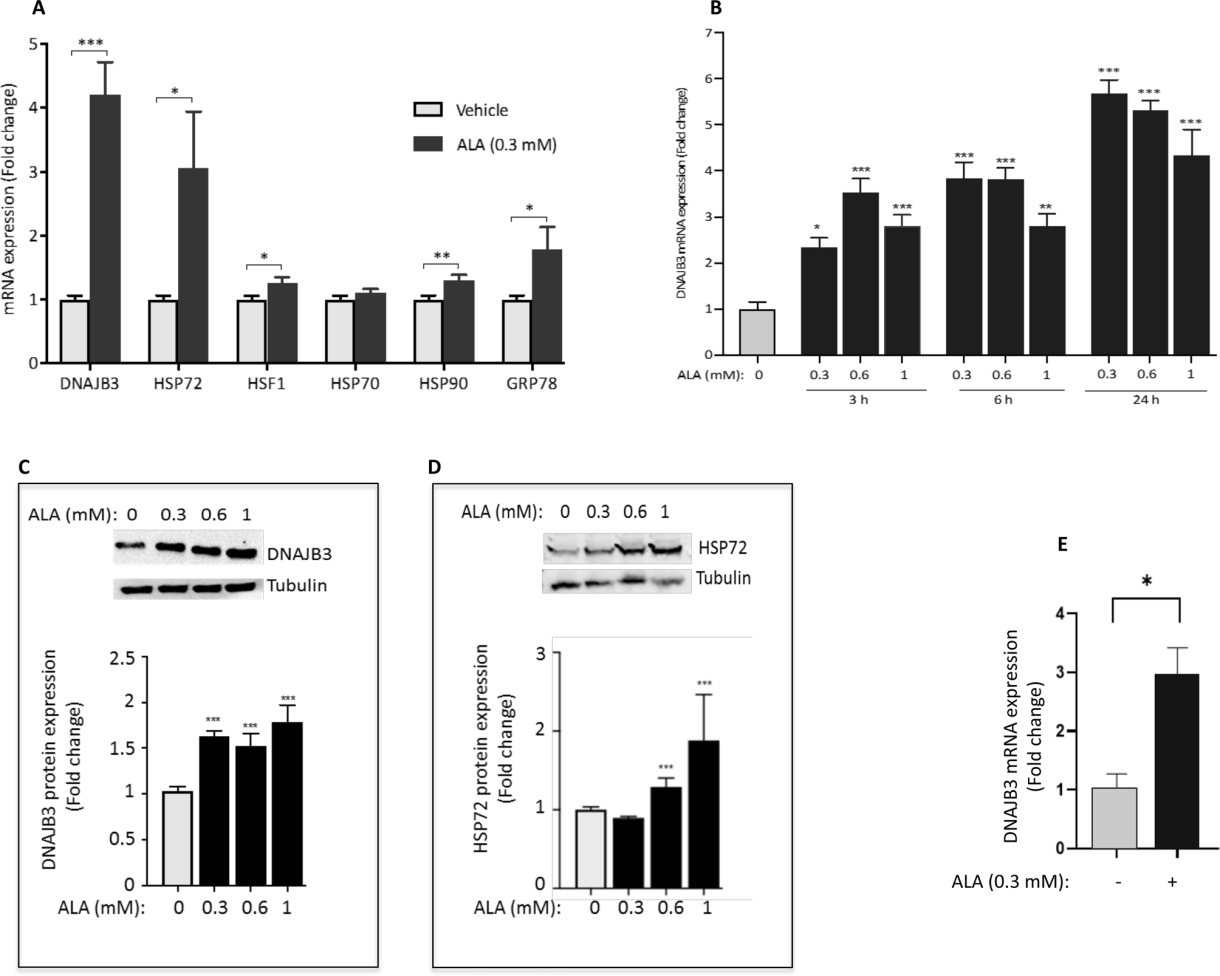
Alpha lipoic acid (ALA) induces the endogenous expression of DNAJB3 in C2C12 and HepG2 cells. (**A**) RT-PCR data showing the effect of 0.3 mM ALA for 24 h on the expression of representative components of the heat shock response in C2C12 cells. (**B**) Dose and time effects of ALA on the expression of DNAJB3 mRNA in C2C12 cells. Western blots confirming the positive effect of 24 h treatment with ALA on the expression of DNAJB3 (**C**) and HSP72 (**D**) proteins in C2C12 cells. Full-length blots are displayed in Supplementary Fig. S1. (**E**) ALA at 0.3 mM for 24 h also increases the expression of DNAJB3 mRNA in HepG2 cells. Ethanol was used at 0.25% as a vehicle. After performing Shapiro–Wilk normality test, *t* test was used to compare the difference between ALA and vehicle. *NS* not significant; *P < 0.05; **P < 0.01; ***P < 0.001.

It has been shown previously that ALA mediates its beneficial effects by activating the HSR. However, these investigations were focused on a limited set of HSPs; namely HSP25, HSP60, HSP72, HSF1 and GRP75^27,33,34^. Our previous in vivo and in vitro investigations confirmed a novel role of DNAJB3 in reducing metabolic stress, improving insulin signaling and promoting glucose uptake^29,30^. These findings suggest that DNAJB3 may represent a relevant therapeutic target against IR and T2D. We therefore decided to assess the effect of ALA on the endogenous expression of DNAJB3 together with other key representative genes of the HSR. Data displayed in Fig. 1A confirmed indeed a significant increase (at least fourfold increase) in the expression of DNAJB3 mRNA in C2C12 cells in response to treatment with 0.3 mM ALA (P < 0.001). Under the same experimental conditions, we confirmed the positive effect of ALA on modulating the expression of other heat shock related genes, although with different degrees, but the highest induction was observed for DNAJB3, followed by HSP72 (Fig. 1A; P < 0.05). We next did a time course and a dose response with ALA and monitored the expression of DNAJB3 mRNA and protein. Data displayed in Fig. 1B,C indicate that ALA induces the expression of DNAJB3 mRNA in a time course-dependent manner (Fig. 1B). Consistent with this finding, ALA triggered also a significant increase in the expression of DNAJB3 protein (Fig. 1C; P < 0.01). The effect of ALA on the expression of HSP72 protein has also been confirmed by western blot analysis (Fig. 1D). To determine whether the effect of ALA on DNAJB3 was cell specific (i.e., C2C12 cells) or not, we examined its effect on liver cell line (HepG2 cells), and observed a significant induction of DNAJB3 mRNA (Fig. 1E; P < 0.05). Finally, we compared the magnitude of ALA effect on the expression of DNAJB3 and HSP72 with heat shock treatment and the results are shown in Supplementary Figure S2. As indicated, heat treatment for 1 h at 43 °C followed by a 4 h recovery at 37 °C resulted in a similar increase in the expression of DNAJB3 mRNA as compared to ALA (P < 0.001). The expression of HSP72 was also increased, albeit to a lower level than that caused by heat (more than 30-fold) in response to heat shock than to ALA treatment (P < 0.01). Taken together, our data indicate clearly that ALA modulates positively the endogenous expression of DNAJB3 both at mRNA and protein levels.

### Pre-treatment of C2C12 cells with ALA alleviates tunicamycin-induced ER stress

**Figure 2:**
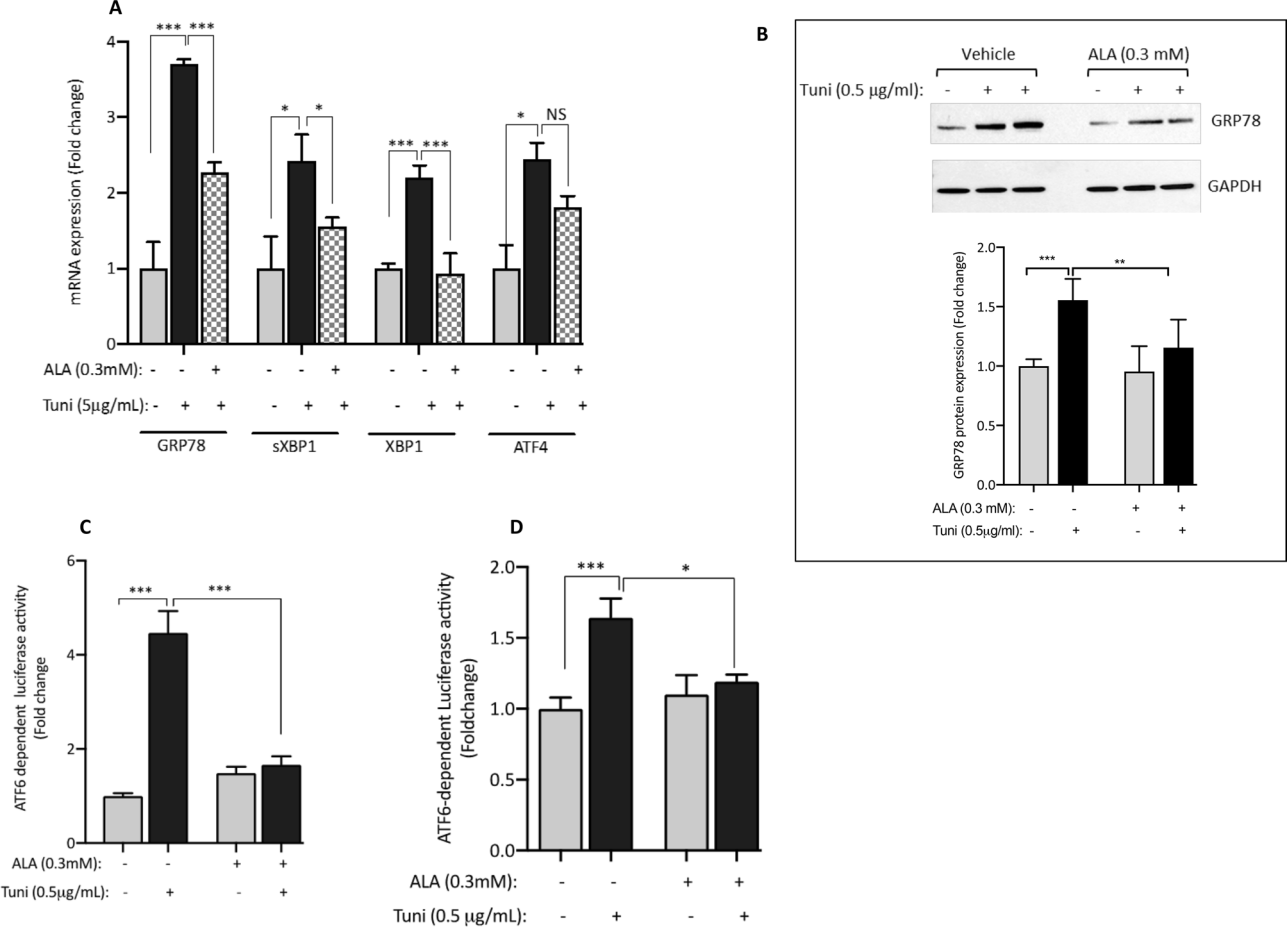
ALA alleviates tunicamycin-induced ER stress. (**A**,**B**) Pre-treatment of C2C12 cells with 0.3 mM ALA abolishes significantly the mRNA expression of classical ER stress markers in response to tunicamycin treatment (**A**). (**B**) Western blot confirming the effect of ALA on tunicamycin-induced expression of GRP78 protein. Full-length blots are displayed in Supplementary Fig. S2. ALA also reduces ATF6-dependent luciferase activity in response to tunicamycin using a functional luciferase-based assay both in C2C12 (**C**) and HepG2 (**D**) cells. Ethanol and DMSO were used at 0.25% as vehicles for ALA and tunicamycin, respectively. After performing Shapiro–Wilk normality test, one way ANOVA was used to compare the effect of treatments. *NS* not significant; *P < 0.05; **P < 0.01; ***P < 0.001.

The contribution of persistent ER stress to the pathogenesis of IR and T2D is well established^35,36^. Several studies reported the effectiveness of ALA in alleviating ER stress^37,38^; however, none of these studies examined the effect of ALA in skeletal muscle cells. We therefore investigated whether pre-treatment of C2C12 cells with ALA could mitigate tunicamycin-induced ER stress by measuring the expression and activity of known ER stress markers. Data displayed in Fig. 2 indicate that overnight treatment of C2C12 cells with 0.5 µg/ml tunicamycin led to a marked increase in the endogenous expression of GRP78, XBP1 and it spliced form sXBP1 and ATF4 as compared to the vehicle (Fig. 2A). In cells pre-treated with ALA, there was a significant reduction in tunicamycin-mediated effect on the expression of most of those markers (Fig. 2A). At the protein level, we confirmed the inhibitory effect of ALA on tunicamycin-induced activation of GRP78 protein (Fig. 2B). To complement these findings, we used a functional luciferase-based assay to examine the effect of ALA on the activity of ATF6 in response to tunicamycin. As shown in Fig. 2C, a fivefold increase in ATF6-dependent luciferase activity in response to 0.5 µg/ml tunicamycin was consistently observed. Pre-treatment of cells with ALA reduced significantly the ATF6-dependent luciferase activity (Fig. 2C; P < 0.001). In HepG2 cells, a similar pattern was also observed (Fig. 2D), although the magnitude of tunicamycin effect was less pronounced than in C2C12. Taken together, these results confirm the beneficial role of ALA in attenuating tunicamycin-induced ER stress in skeletal muscle and in liver cells.

### ALA stimulates the expression of mitochondrial markers and the oxidative stress scavenging system in C2C12 cells

**Figure 3:**
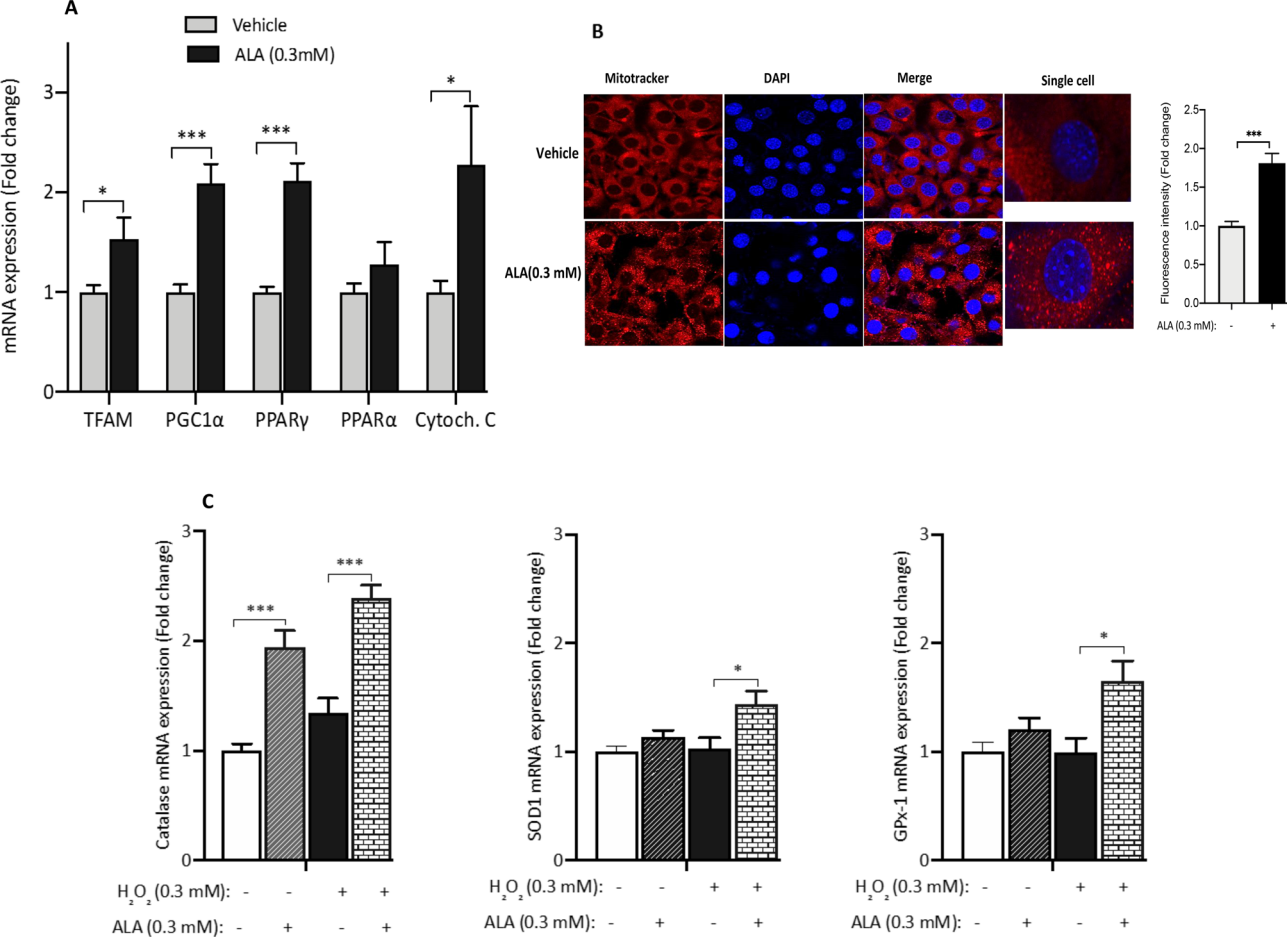
ALA improves mitochondrial function and the oxidative stress scavenging system. (**A**) ALA treatment triggers a significant increase in the expression of genes involved in mitochondrial biogenesis and function. (**B**) Representative images showing the effect of ALA treatment on enhancing the mitochondrial activity as revealed by MitoTracker (Red staining). Cells were counter screened with DAP (Blue staining). The images were acquired using a 25 ×/0.8 NA objective lens (LD LCI Plan‐Apochromat; Carl Zeiss Inc.,) mounted on a LSM (Zeiss LSM 780; Carl Zeiss Inc.). The fluorescence intensity (40 cells in the vehicle group and 41 cells in the ALA group) was quantified using ImageJ 1.52v software (NIH, Bethesda, MA, USA) and the fold change in fluorescence intensity was calculated and plotted. (**C**) ALA stimulates the endogenous mRNA expression of Catalase, Superoxide dismutase 1 (SOD1) and glutathione peroxidase 1 (GPX1) genes in response to 300 μM H_2_O_2_ treatment for 3 h. Ethanol was used at 0.25% as vehicles for ALA. After performing Shapiro–Wilk normality test, *t* test was used to compare the difference between ALA and vehicle (**A**) and one-way ANOVA was used to compare the effect of treatments (**C**). *NS* not significant; *P < 0.05, **P < 0.01. ***P < 0.001.

Dysfunction of mitochondria and/or its biogenesis was linked to the pathogenesis of IR and T2D^7,39–41^. One of the attractive features of ALA is its effect on promoting mitochondrial function and biogenesis^14,42,43^. We measured the expression of key representative mitochondrial marker genes in C2C12 in response to ALA. As shown in Fig. 3A, ALA treatment triggers a significant increase in the expression of TFAM (P < 0.05), PPARγ and its coactivator PGC1α (P < 0.001), and cytochrome C (P < 0.05). No significant effect was observed for PPARα (Fig. 3A). To complement these data, we examined the status of mitochondria in response to ALA by confocal microscopy using MitoTracker staining kit. Representative images showed an increase of active mitochondria following ALA treatment as compared to the vehicle (Fig. 3B); confirming thus the above mRNA data.

We next examined the effect of ALA on modulating the mRNA expression of genes encoding for representative antioxidant enzymes; namely catalase, glutathione peroxidase (GPX1) and superoxide dismutase 1 (SOD1). Results displayed in Fig. 3C indicate a significant increase in the mRNA expression levels of catalase (P < 0.001) both at basal level and after H_2_O_2_ treatment. A positive effect of ALA on the expression of SOD1 and GPX1 genes was observed but only under oxidative stress conditions (Fig. 3C; P < 0.05).

### Silencing the expression of DNAJB3 abolished the beneficial effect of ALA on alleviating tunicamycin-induced ER stress

**Figure 4:**
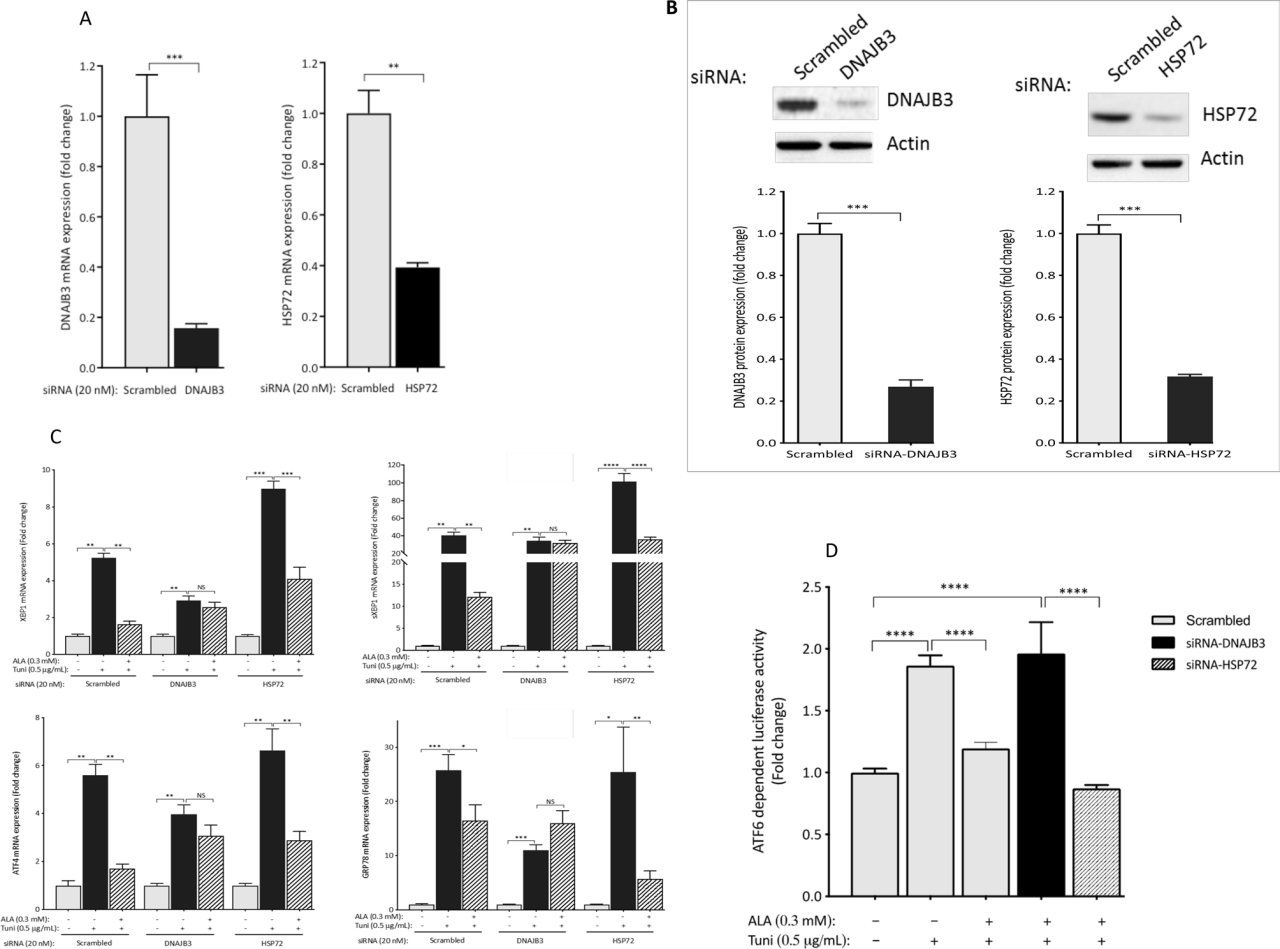
Silencing the expression of DNAB3 abolishes the protective effect of ALA on tunicamycin-induced ER stress in C2C12 cells. (**A**,**B**) Knocking down the expression of DNAJB3 and HSP72 with 20 nM of specific siRNA blunted the endogenous expression of DNAJB3 and HSP72 mRNAs (**A**) and proteins (**B**). Actin was used as internal control for both RT-PCR and western blots. Full-length blots are displayed in Supplementary Fig. S4. ALA fails to protect siRNA DNAJB3-transfected C2C12 cells from tunicamycin-induced mRNA expression of ER stress markers (**C**) and ATF6-dependent luciferase activity (**D**). Ethanol and DMSO were used at 0.25% as vehicles for ALA and tunicamycin, respectively. After performing Shapiro–Wilk normality test, *t* test was used to compare the difference between ALA and vehicle (**A**) and two-ways ANOVA was used to compare the effect of treatments (**B**,**C**). *NS* not significant; *P < 0.05; **P < 0.01; ***P < 0.001; ^#^P < 0.0001.

**Figure 5:**
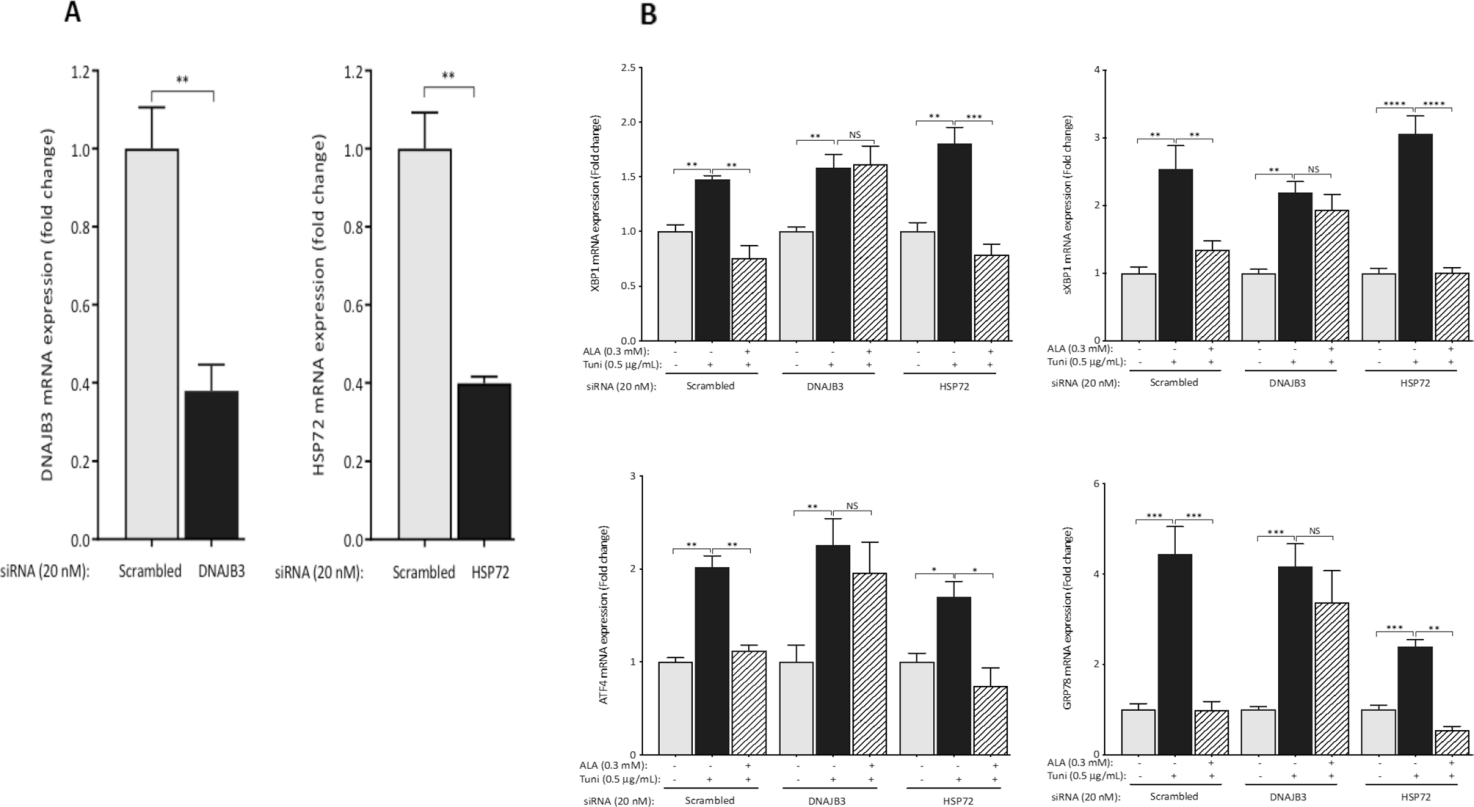
Silencing the expression of DNAB3 abolishes the protective effect of ALA on tunicamycin-induced ER stress in HepG2 cells. (**A**) Knocking down the expression of DNAJB3 and HSP72 mRNA with 20 nM of specific siRNA reduced significantly the endogenous expression of DNAJB3 and HSP72 mRNA. Actin gene was used as a reference control. ALA fails to protect cells from tunicamycin-induced mRNA expression of ER stress markers (**B**) and ATF6-dependent luciferase activity (**C**) in cells transfected with siRNA specific for DNAJB3. Ethanol and DMSO were used at 0.25% as vehicles for ALA and tunicamycin, respectively. After performing Shapiro–Wilk normality test, *t* test was used to compare the difference between ALA and vehicle (**A**) and two-ways ANOVA was used to compare the effect of treatments (**B**,**C**). *NS* not significant; *P < 0.05; **P < 0.01; ***P < 0.001; ^#^P < 0.0001.

We have previously reported a negative regulation of DNAJB3 expression when ER stress is induced^28^. Reciprocally, both basal and tunicamycin-induced ER stress are significantly reduced when DNAJB3 is overexpressed^30^. This is suggestive of a mutual negative feedback regulation between DNAJB3 and ER stress. To get more insight in the possible role of DNAJB3 as a molecular determinant through which ALA mediates its beneficial effect on ER stress, we knocked down the expression of DNAJB3 using siRNA and then, induced ER stress with tunicamycin in cells pre-treated with ALA. As controls, we used scrambled siRNA (negative control) and siRNA targeting HSP72. As shown in Fig. 4A, silencing the expression of DNAJB3 and HSP72 in C2C12 cells with specific siRNA abrogated effectively the expression of DNAJB3 and HSP72 mRNA by 90% (P < 0.001) and 65% (P < 0.01), respectively despite the same transfection protocol. At protein level, the knock down effect on the endogenous expression of DNAJB3 and HSP72 was monitored by western blots. As shown in Fig. 4B, silencing the expression of DNAJB3 and HSP72 resulted in a marked decrease in their protein levels, thus confirming the RT-PCR data. The ability of ALA to reduce ER stress when the expression of DNAJB3 is silenced is illustrated in Fig. 4C,D. As shown, DNAJB3 siRNA abolished the beneficial effect of ALA on attenuating tunicamycin-induced ER stress marker genes as compared to HSP72 and scrambled siRNA (Fig. 4C). Consistent with these findings, ALA treatment significantly reduced the ATF6-dependent luciferase activity in response to tunicamycin as compared to scrambled and HSP72 siRNA (Fig. 4D; P < 0.01). The finding that ALA stimulates the expression of DNAJB3 in HepG2 (Fig. 1E) prompted us to investigate whether DNAJB3 could also mediate the beneficial effect of ALA on alleviating ER stress in liver cells. We first examined the efficiency of siRNA in silencing the expression of their target genes and the data is illustrated in Fig. 5A. As shown, the mRNA expression of both genes was reduced by more than 60% (P < 0.01). We then assessed the ability of ALA to reduce ER stress triggered with tunicamycin. Data displayed in Fig. 5B confirmed the role of DNAJB3 but not HSP72 in mediating the effect of ALA in alleviating ER stress in liver cells. Together, these findings suggest that ALA attenuates ER stress, at least in part through DNAJB3.

### ALA-stimulated glucose uptake is significantly reduced in cells transfected with DNAJB3-siRNA

**Figure 6:**
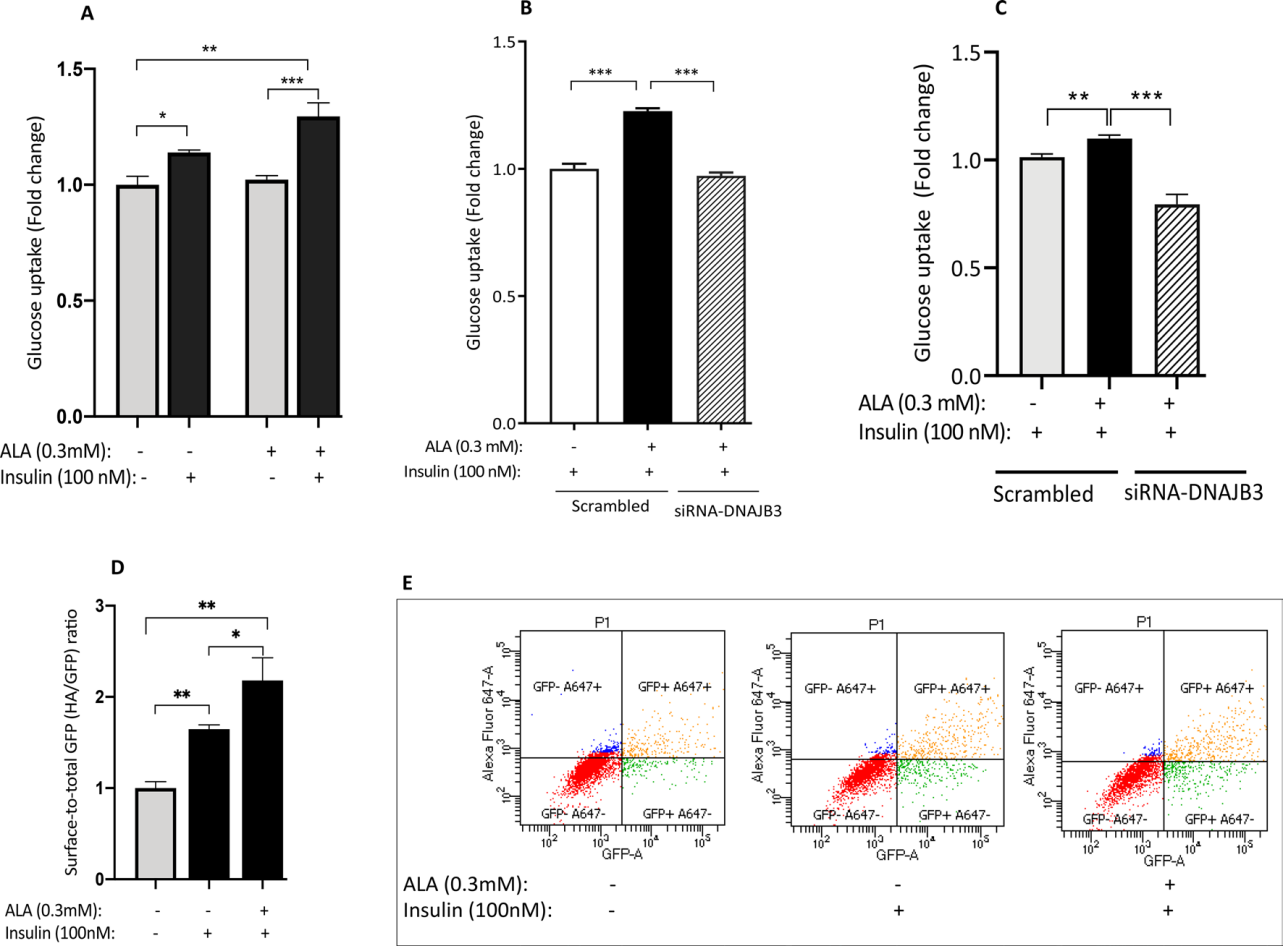
Silencing the expression of DNAB3 abrogated the effect of ALA on enhancing glucose uptake in C2C12 and HepG2 cells. (**A**) Effect of ALA on insulin-stimulated glucose uptake in C2C12 cells. (**B**,**C**) Silencing the expression of DNAJB3 abrogated the effect of ALA on insulin-stimulated glucose uptake as compared to scrambled siRNA control in C2C12 cells (**B**) and HepG2 cells (**C**). (**D**,**E**) Effect of ALA on insulin-mediated Glut4 translocation in C2C12 cells. After performing Shapiro–Wilk normality test, *t* test was used to compare the difference between ALA and vehicle (**A**,**D**) and one-ways ANOVA was used to test the DNAJB3 effect (**B**,**C**). Ethanol was used at 0.25% as vehicles for ALA. *NS* not significant; **P < 0.01; ***P < 0.001.

In our previous findings, we reported that DNAJB3 has a positive effect on enhancing both basal and insulin-stimulated glucose uptake^29,30^. In this investigation, we assessed first the effect of ALA on glucose uptake in C2C12 and then, investigated whether DNAJB3 is involved in such effect. As displayed in Fig. 6A, in the absence of ALA, insulin significantly increased glucose uptake (as measured by accumulation of 2-NBDG, a fluorescently-labeled deoxyglucose analog in cells); when cells were pre-treated with ALA, the effect of insulin on glucose uptake was further increased. To complement this finding, we monitored Glut4 translocation to the plasma membrane using pHA-Glut4-GFP expression vector. Flow cytometry data revealed a positive effect of ALA on enhancing insulin-stimulated Glut4 translocation to the plasma membrane (P < 0.01; Fig. 6D,E); thus supporting the glucose uptake findings. Under the conditions where the expression of DNAJB3 has been silenced, ALA failed to promote insulin-stimulated glucose uptake both in C2C12 cells (Fig. 6B; P < 0.001) and HepG2 cells (Fig. 6C).

## Discussion

Impaired expression of HSP25, HSP72 and DNAJB3; three important components of the HSR has been reported to negatively correlate with the degree of IR^28,44,45^. Consistent with this, interventions that activate the HSR; irrespective of the means to achieve it (i.e., heat therapy, physical exercise, mild electrical therapy, genetic overexpression and pharmacological drugs) showed a greater improvement in insulin sensitivity and glucose homeostasis^28,46–49^. Recent studies from our group demonstrated a clear physiological role of DNAJB3 in mitigating metabolic stress and improving insulin signaling and glucose homeostasis in C2C12 skeletal muscle cells and 3T3-L1 adipocytes^29,30^. In rat model of high fat-induced IR and glucose intolerance, the ability of ALA to prevent the decrease of HSP25 and HSP72 levels with effective improvement of insulin action and glycemic index has been reported^34^. In the present study, we investigated the effect of ALA on the endogenous expression of DNAJB3 in metabolically relevant cells and the significance of such effect on ER stress and glucose uptake. Using C2C12 and HepG2 cell lines, we provide evidence that ALA treatment: (1) induces the expression of DNAJB3, (2) alleviates ER stress triggered by tunicamycin and enhances insulin-stimulated glucose uptake and Glut4 translocation and, (3) knocking down the expression of DNAJB3 with siRNA abolished the beneficial effect of ALA in alleviating tunicamycin-induced ER stress and enhancing insulin-stimulated glucose uptake. Taken together, this study is the first to show DNAJB3 as a molecular determinant that mediates the beneficial effect of ALA in attenuating metabolic stress. The identification of DNAJB3 as a downstream target of ALA supports further the importance of the HSR in mitigating the key drivers of IR.

DNAJB3; previously known as MSJ-1 was initially described in mice as a gene involved in male reproduction^50^, but given the presence of a highly conserved and functionally critical J-domain, the gene was subsequently named DNAJB3; a member of the DNAJ/HSP40 cochaperone family that acts as an obligate partner and critical regulator of the activity and substrate specificity of HSP70 chaperone^51^. DNAJB members exert their role by stimulating the ATPase activity of HSP70 through their J-domain, thereby keeping the bound substrates in successive refolding cycles^52,53^. Recently, our group was the first to demonstrate a novel in vitro role of DNAJB3 in insulin signaling and glucose metabolism^29,30^. Based on this novel role, and given its importance in regulating the chaperone activity of HSP70; DNAJB3 represents a potential therapeutic target for diseases associated with IR and proteotoxicity. The findings of the current study; despite been carried out in vitro, is the first proof-of-concept showing the druggability of DNAJB3 target with a relevant pharmacological drug and provide rationale for further in vivo follow-up investigations. More particularly, the validation of ALA effect on the expression of DNAJB3 in a mouse model of high fat-induced IR remains to be confirmed.

In all living organisms, cells cope with various noxious conditions by inducing a dedicated set of anti-stress responses to ensure normal cellular homeostasis and any persistent dysregulation of these responses may lead to pathological disorders. The detrimental consequences of impaired HSR on the pathogenesis of IR and T2D are well established^8,54^. This has been particularly the case for HSP25^55^, HSP72^56^, and DNAJB3^28,29^. Given their ability to bind and inactivate JNK1 and IKKβ kinases, HSPs emerged as attractive therapeutic targets for obesity-induced IR and T2D. Previous studies showed positive effect of ALA on modulating various components of the HSR such as HSP25, HSP72 and HSF1. However, this current study is the first to report the effect of ALA effect on DNAJB3 expression. ALA treatment caused significant increase in DNAJB3 expression in C2C12 and HepG2 cells. The question that arises from this investigation is how ALA induces the expression of DNAJB3. The most likely mechanism for HSPs induction would be activation of HSF1; the master transcription factor controlling the HSR. Under our experimental conditions, we observed a slight, but significant increase in HSF1 mRNA levels in response to ALA (Fig. 1A) and this could play a role in DNAJB3 activation. Similar findings have been reported in streptozotocin-induced diabetic rat in response to ALA^27^. By contrast, ALA supplementation showed no effect on HSF1 expression in the skeletal muscle of high fat fed rat model of IR^34^, as well as under heat stress conditions in Caco-2 cells^57^. The dose, route of administration, biological context and duration of treatment could explain the discrepancy between those studies. Moreover, the DNA-binding activity of HSF1 has been shown to be potentiated with SIRT1 deacetylase^58^. Interestingly, SIRT1 expression is also positively regulated with ALA^59^. Alternatively, ALA could control the expression through the nuclear factor-erythroid 2 (Nrf2); another safeguard transcription factor that regulates the expression of anti-oxidant response genes^60^, as well as HSR genes^61^. In high fat diet-induced hepatic steatosis rat Goto-Kakizaki model, ALA prevented hepatic steatosis by incrementing antioxidant defense systems through Nrf2^62^. Nrf2 executes its task by binding to the antioxidant response element (ARE) in the promoter region of its downstream target genes and subsequently drives their expression^63^. Interestingly, both mouse and human DNAJB3 promoters have a putative ARE. Preliminary data show increased expression in DNAJB3 mRNA and protein in response to sulforaphane and resveratrol, two potent activators of Nrf2 (data not shown). Whether Nrf2 is involved in the activation of DNAJB3 in response to ALA remains to be investigated.

Another important finding of this investigation is the effect of ALA on tunicamycin-induced ER stress; a major trigger of IR and T2D^64,65^. The ER is a dispersed organelle throughout the cell that performs important homeostatic functions related to proteostasis, lipid metabolism and calcium storage^66^. These processes rely on the protein folding activity of chaperone machinery densely populated in the ER. Disruption of ER homeostasis occurs when the folding capacity of the ER fails to accommodate the overwhelming load of misfolded and unfolded proteins, leading thus to ER stress. This elicits a potent adaptive response known as the unfolded protein response (UPR); an acute mechanism that assists in restoring ER activity and reestablishing cellular homeostasis. However, sustained chronic UPR activation as a result of persistent (unresolved) ER stress has been implicated in a variety of metabolic disorders, including obesity, IR and T2D^35,36,67^. By contrast, restoring ER homoeostasis either pharmacologically or genetically was shown to reverse IR and improve glucose homeostasis^68,69^. In eukaryotic cells, the UPR is initiated by the activation of three ER stress canonical transducers that act in concert to restore ER homeostasis: PKR-like ER kinase (PERK), inositol-requiring enzyme-1α (IRE1α), and activating transcription factor-6 (ATF6). Under normal conditions, the luminal stress-sensing domain of PERK, IRE1α, and ATF6 interacts with GRP78 chaperone, however upon accumulation of unfolded proteins, GRP78 dissociates from these transducers, leading to their activation^70^. Each of these transducers activates specific pathways and collectively leads to decreased overall protein synthesis, enhanced ER folding capacity and increased degradation of misfolded proteins, resulting in either recovery of ER homeostasis or cell death^71^. Tunicamycin is a well-known ER stress inducer both in vitro and in vivo. Our results show that tunicamycin leads to a marked increase in the expression of the genes of the UPR system, namely GRP78, ATF4, XBP and its spliced form sXBP1 (Fig. 2). Consistent with this, we observed also an increase in the luciferase activity driven by ATF6 in response to tunicamycin (Fig. 2). ATF6 activation along with GRP78, ATF4, XBP1 and sXBP1 up-regulation following low dose (0.5 µg/ml) tunicamycin treatment for a relatively short term (i.e., overnight) is likely to trigger an adaptive response rather than an apoptotic response^72^. In the current study, we did not investigate the status of apoptosis in response to 0.5 µg/ml Tunicamycin for overnight, however, a most recent study demonstrated that apoptotic pathway becomes activated only when the ER stress is sustained (≥ 72 h following tunicamycin treatment)^73^.

Interestingly, we found in this investigation that tunicamycin-induced increase in GRP78, ATF4, XBP and sXBP1s mRNA expression was impeded by ALA (Fig. 2). At the protein level, we only measured the effect of ALA on tunicamycin-mediated expression of GRP78 protein and confirmed the mRNA data (Fig. 2B). In functional assays, tunicamycin-mediated ATF6 activation was significantly reduced with ALA (Fig. 2C,D). Our results showing effective attenuation of ER stress with ALA are consistent with several previous in vivo and in vitro studies^37,38,74^.

The most important finding in our current investigation is the demonstration that DNAJB3 is one of the molecular targets through which ALA attenuates ER stress. Defects in the HSR and persistent ER stress are one of the critical aberrations underlying IR and T2D. Alleviation of ER stress and enhancement of the HSR have previously been considered as attractive potential therapeutic pathways against several chronic diseases^49,56,75^. In L6 cells, the ability of ALA to prevent anisomycin-mediated JNK activation is abolished upon inhibition of the HSR with KNK437 drug, supporting thus a role of the HSR as mediator of the metabolic actions elicited by ALA^34^. We have previously reported the negative regulation of DNAJB3 expression when ER stress is induced^28^. However, the molecular mechanism by which ER stress downregulates the expression of DNAJB3 remains to be fully elucidated. More recently, we showed a reciprocal effect of DNAJB3 on both basal and tunicamycin-induced ER stress^30^. This is suggestive of a mutual negative feedback regulation between DNAJB3 and ER stress that provides a functional crosstalk between both stress pathways. To get more insight into the possible role of DNAJB3 as a molecular determinant through which ALA alleviates ER stress, we silenced the expression of DNAJB3 using siRNA and then, assessed the ability of ALA to reduce ER stress in response to tunicamycin. Our data show that DNAJB3 siRNA but not HSP72 abolished the beneficial effect of ALA on tunicamycin-induced ER stress marker genes as compared to HSP72 siRNA and scrambled siRNA control (Fig. 4C; P < 0.05). Consistent with these findings, the effect of ALA on reducing ATF6 activity in response to tunicamycin was also reduced significantly when DNAJB3 is specifically silenced (Fig. 4D; P < 0.05). Together, these findings suggest that ALA attenuates ER stress, at least in part through DNAJB3.

It is not clear whether DNAJB3 exerts a direct effect on ER stress or indirectly via other pathways. The close link between ER stress and mitochondrial homeostasis has been well described^76,77^. In HepG2 cells, ALA prevented ER stress-induced IR by enhancing mitochondrial function, but surpisingly; it failed to abrogate the expression of tunicamycin-induced ER stress markers^74^. In our previous study, we observed a positive correlation between DNAJB3 levels and maximum oxygen consumption in human subjects^28^. In C2C2 cells, overexpression of DNAJB3 stimulated the expression of conventional markers of mitochondrial biogenesis and function such as TFAM, PGC1α, PPARγ and OXPHOS subunits (unpublished data). Interestingly, this pattern was also observed in the current study. Specifically ALA supplementation increased the mRNA levels of TFAM, PGC1α, PPARγ and cytochrome C in C2C12 cells (Fig. 3A) and enhanced mitochondrial activity as revealed by MitoTracker staining using confocal microscopy (Fig. 3B); consistent with previous findings in other cellular systems^78^. It is possible that DNAJB3 attenuates ER stress by promoting mitochondrial homeostasis. In this context, it will be important to assess the effect of DNAJB3 on ER stress under the conditions where the mitochondrial function is impaired (i.e., oligomycin). Alternatively, DNAJB3 could physically interact with one or several transducers of the UPR and trigger an inhibitory effect on their respective activities. This has been particularly the case for ERdj4/DNAJB9; another member of DNAJB family that prevents the oligomerization of IRE1α transducer by promoting complex formation between the luminal GRP78 and luminal stress-sensing domain of IRE1α and thereby repressing its enzymatic activity^79^. Undoubtedly further more in-depth studies are needed to investigate the possible physical interaction between DNAJB3 and different transducers of the UPR as well as the functional consequences.

ALA may also attenuate ER stress via reduction of the oxidative stress through stimulating the anti-oxidant response genes as previously reported^80^. ER stress and oxidative stress are important mechanisms of IR. We have recently reported the ability of DNAJB3 to mitigate oxidative stress induced with H_2_O_2_^30^_._ In the current study, ALA treatment stimulated the expression of the oxidative stress scavenging genes Catalase, SOD1 and GPx-1 in response to H_2_O_2_ (Fig. 3C), replicating previous findings in C2C12 cells^80^. To investigate whether the beneficial effects of ALA on ER stress and oxidative stress is associated with improved glucose and insulin metabolism, C2C12 cells were treated with ALA and insulin-induced glucose uptake was performed. Our results showed a significant increase in insulin-stimulated glucose uptake following ALA treatment, similar to a pattern observed with DNAJB3 overexpression in C2C12 cells. It is well established that both mitochondrial dysfunction and ER stress are involved in IR and impairment of glucose metabolism^81^. The mitochondria and ER are physically and functionally interconnected^82^. With respect to metabolic diseases, whether dysfunction of mitochondria is a trigger of ER stress or vice-versa is still poorly understood^83,84^. This suggests that the beneficial effect of ALA on insulin-stimulated glucose uptake is likelihood to be mediated through either improvement of mitochondrial function or through suppressing ER stress or through both. Further investigations are needed to elucidate the molecular mechanisms underlying the beneficial effect of ALA.

To examine the specificity of DNAJB3 on the effect of ALA on insulin-stimulated glucose uptake, we silenced the expression of DNAJB3 using siRNA and then, assessed the effect of ALA on glucose uptake. Our data show that inhibition of DNAJB3 abolished the beneficial effect of ALA on insulin-stimulated glucose uptake in both C2C12 and HepG2 cells; suggesting that ALA improves glucose uptake through DNAJB3.

In summary, findings from this study show for the first time a stimulatory effect of ALA on the expression of DNAJB3 in C2C12 and HepG2. More importantly, we provide a novel mechanism by which ALA modulates ER stress and glucose uptake. Our data warrant future investigations in experimental animal models of high fat-induced IR and T2D in wild type and DNAJB3 KO mice to confirm the in vivo effect of ALA on activating the expression of DNAJB3 and protecting against the metabolic adverse effects associated with high fat diet.

## Supplementary information

Supplementary Figures.
