# Supplementary material for: Alpha lipoic acid attenuates ER stress and improves glucose uptake through DNAJB3 cochaperone
Source: Sci Rep. 2020 Nov 24;10:20482. doi: 10.1038/s41598-020-77621-x (PMC7687893; doi:10.1038/s41598-020-77621-x)
Supplement: Supplementary file 1 — Supplementary Figures. [file 41598_2020_77621_MOESM1_ESM.pdf]

Supplementary information

Alpha Lipoic acid attenuates ER stress and improves glucose uptake through DNAJB3 cochaperone

Abdoulaye Diane[1], Naela Mahmoud[1,2], Ilham Bensmail[1], Namat Khattab[1], Hanan A. Abunada[1] and Mohammed Dehbi[1,2]*

[1]Diabetes Research Center, Qatar Biomedical Research Institute, Hamad Bin Khalifa University, Qatar Foundation, Doha, Qatar

[2]College of Health and Life Sciences, Hamad Bin Khalifa University, Qatar Foundation, Doha, Qatar

*Corresponding author: Mohammed Dehbi, mdehbi@hbku.edu.qa

Qatar Biomedical Research Institute, Doha, Qatar; P.O. Box: 34110; Doha - Qatar

Tel: +974 4454 6339

Supplementary Figure S1

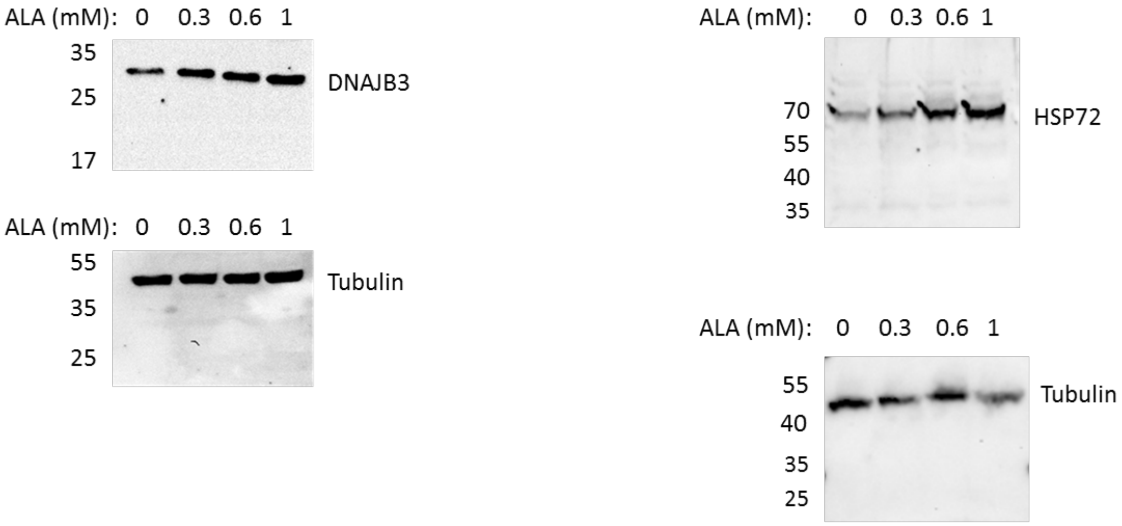

Supplementary Figure S1: Alpha lipoic acid (ALA) induces the endogenous expression of DNAJB3 and HSP72 proteins in C2C12 cells as revealed by western blots. Cells were treated for 24h with increasing amounts of ALA. γ-Tubulin was used as internal control.

Supplementary Figure S2

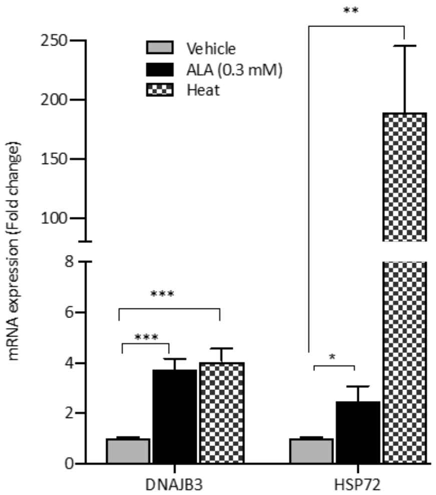

Supplementary Figure S2: Effect of ALA and heat shock treatment on the endogenous expression of DNAJB3 and HSP72 mRNA as revealed by RT-PCR. Heat shock was induced by incubating cells at 43°C for 1h followed by a 4h recovery at 37°C and then harvested.

Supplementary Figure S3

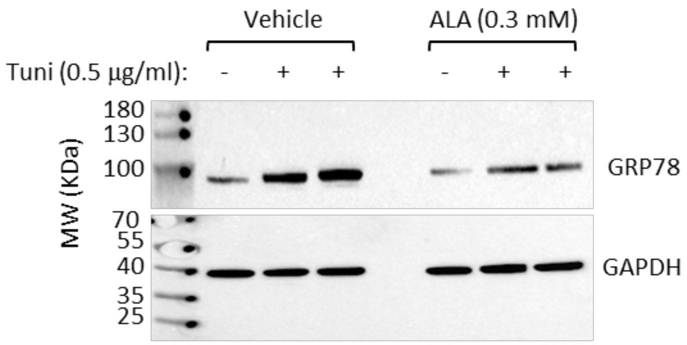

Supplementary Figure S3: ALA alleviates tunicamycin-induced expression of GRP78 protein in C2C12 cells as monitored by western blot. GAPDH was used as internal control.

Supplementary Figure S4

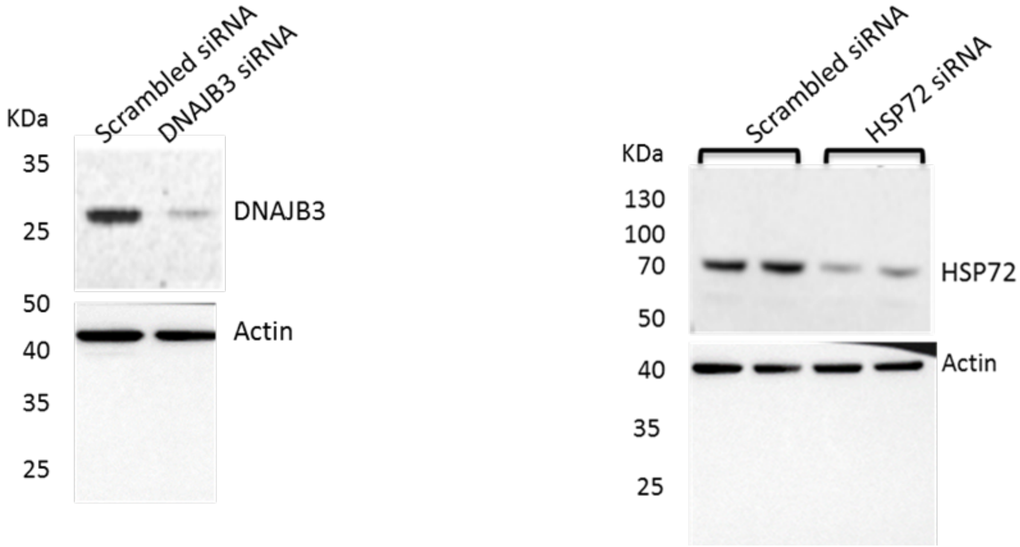

Supplementary Figure S4: Western blots showing the effect of DNAJB3 and HSP72 siRNAs on the endogenous expression of their target proteins. Scrambled siRNA was used as a negative control.
